# Multi-Modal Microfluidics (M^3^) for Sample Preparation of Liquid Biopsy: Bridging the Gap between Proof-of-Concept Demonstrations and Practical Applications

**DOI:** 10.3390/mi13020209

**Published:** 2022-01-28

**Authors:** Yaoping Liu, Wei Wang

**Affiliations:** 1 School of Integrated Circuits, Peking University, Beijing 100871, China; yaopingliu@pku.edu.cn or; 2 Antimicrobial Resistance (AMR) and Critical Analytics for Manufacturing Personalized-Medicine (CAMP) IRGs, Singapore-MIT Alliance for Research and Technology (SMART) Centre, Singapore 138602, Singapore; 3 National Key Laboratory of Science and Technology on Micro/Nano Fabrication, Beijing 100871, China; 4 Frontiers Science Center for Nano-Optoelectronics, Peking University, Beijing 100871, China

**Keywords:** liquid biopsy, sample preparation, microfluidics

## Abstract

Liquid biopsy, the technique used to shed light on diseases via liquid samples, has displayed various advantages, including minimal invasiveness, low risk, and ease of multiple sampling for dynamic monitoring, and has drawn extensive attention from multidisciplinary fields in the past decade. With the rapid development of microfluidics, it has been possible to manipulate targets of interest including cells, microorganisms, and exosomes at a single number level, which dramatically promotes the characterization and analysis of disease-related markers, and thus improves the capability of liquid biopsy. However, when lab-ready techniques transfer into hospital-applicable tools, they still face a big challenge in processing raw clinical specimens, which are usually of a large volume and consist of rare targets drowned in complex backgrounds. Efforts toward the sample preparation of clinical specimens (i.e., recovering/concentrating the rare targets among complex backgrounds from large-volume liquids) are required to bridge the gap between the proof-of-concept demonstrations and practical applications. The throughput, sensitivity, and purity (TSP performance criteria) in sample preparation, i.e., the volume speed in processing liquid samples and the efficiencies of recovering rare targets and depleting the backgrounds, are three key factors requiring careful consideration when implementing microfluidic-based liquid biopsy for clinical practices. Platforms based on a single microfluidic module (single-modal microfluidics) can hardly fulfill all the aforementioned TSP performance criteria in clinical practices, which puts forward an urgent need to combine/couple multiple microfluidic modules into one working system (i.e., multi-modal microfluidics, M^3^) to realize practically applicable techniques for the sample preparation of liquid biopsy. This perspective briefly summarizes the typical microfluidic-based liquid biopsy techniques and discusses potential strategies to develop M^3^ systems for clinical practices of liquid biopsy from the aspect of sample preparation.

Liquid biopsy is the sampling and analysis of physiological fluids (i.e., non-solid biological tissue) to obtain information useful for the diagnosis and monitoring of diseases such as cancer and infection [1,2,3,4,5,6,7,8,9]. Various physiological fluids, such as blood, urine, sputum, saliva, cerebrospinal fluid (CSF), bronchoalveolar lavage fluid (BALF), and pleural effusion, which are collected in routine clinical diagnoses, can be operated on by different liquid biopsy techniques. By liquid biopsy, precise information about diseases can be obtained by separating, detecting, and analyzing a variety of targets of interest, including cells (e.g., circulating tumor cell (CTC), exfoliated tumor cell (ETC), white blood cell (WBC), red blood cell (RBC)) [9,10,11,12,13,14,15,16,17,18,19,20,21], microorganisms (e.g., fungi, bacteria, virus) [5,6,7,22,23,24,25,26], extracellular vesicles (e.g., exosome) [27,28,29,30,31,32], proteins [27,29,33], and nucleic acids [4,29,34]. Liquid biopsy has displayed many advantages, such as minimal invasiveness, low risk, and ease of multiple sampling for dynamic monitoring, and has therefore drawn extensive attention from multidisciplinary fields in the past decade. So far, a variety of techniques for liquid biopsy, especially the platforms based on microfluidics, has been developed [4,5,7,8,10,19,22,23,34,35,36,37].

In practical applications, liquid biopsy requires various procedures, such as sample preparation (i.e., target recovery/preconcentration) and target detection/analysis, which address the demand for multi-functional combinations to realize a sample-to-answer system. As shown in Figure 1, there exists a big gap resulting from a volume mismatch and a concentration mismatch between the raw clinical samples and the detection/analysis platforms. Therefore, as a requisite bridge, sample preparation counts for a lot in high-performance detection/analysis, especially in practices of complex and large-volume clinical specimens. This point can be illustrated with an example of a cancer-targeted liquid biopsy. Although a large number of biomarkers have been discovered in the last few decades, only a handful have been approved by the FDA [33]. It cannot be envisaged that liquid biopsy will replace traditional tissue biopsy, the current gold standard in clinics, in the near future. This is attributed to the lack of specificity (or robustness) in the detection of irregularly appearing biomarkers in heterogeneous physiological samples. The lack of specificity mainly results from how the targets of interest are extremely small in number. For instance, there are too few CTCs to fulfill viable characterizations, there usually being as few as 5–10 CTCs among millions of WBCs per milliliter of whole blood in early-stage cancer patients. It is easy to imagine how hard it is to directly detect/analyze the rare CTCs from huge backgrounds. Therefore, sample preparation for fulfilling the fast processing of large-volume clinical liquids and the high-performance separation of rare targets of interest from complex backgrounds is a requisite.

Microfluidics is poised to impact and transform the biomedical research and clinical detection industry in ways that have never been seen when using conventional methods. In recent years, with the rapid development of microfluidics, it has been possible to manipulate targets of interest, including cells, microorganisms, and exosomes, at a single number level, which dramatically promotes the characterization and analysis of disease-related markers, and thus improves the capability of liquid biopsy [2,4,7,10,22,23,34,35,38]. However, this field is overloaded with interesting academic publications of proof-of-concepts, with their being far less transplantations fulfilling practical applications [36,37]. As microfluidic-based liquid biopsy techniques mature from proof-of-concept demonstrations toward practical applications, several persistent barriers have prevented these techniques from becoming a widespread commodity, even after successful laboratory validation and rounds of commercial investment [8,19,36,37]. A lot of factors result in the aforementioned phenomenon and are usually mainly divided into device (or technique)-related barriers relating to applicability to real clinical samples and the expense of practicality for the end-user and commercialization-related barriers relating to intellectual property and market need [8]. Here, we focus on the discussion of technique-related challenges and potential strategies for bridging the gap between the proof-of-concept demonstrations and practical applications.

In recent years, the integrated microfluidic platforms for multiplex detecting/analyzing targets have attracted widespread attention and been preliminarily developed [4,5,13,30,32,33,38]. Meanwhile, it is noteworthy that the microfluidic-based sample preparation increasingly shows strong competitiveness to be a bigger game-changer compared to the biochemical reagents-based wet process in improving detection/analysis performance (e.g., sensitivity, specificity) [4,5,23,30]. Further, a big issue is that although these platforms perform very well with demo samples in labs, most will fail while transferring them to process raw clinical specimens. Efforts in sample preparation of clinical specimens are urgent for transferring the lab demonstrated techniques to practical applications. The throughput, sensitivity, and purity (TSP performance criteria) in sample preparation, i.e., the volume speed in processing liquid samples and the efficiencies of recovering rare targets and depleting the backgrounds, are three key factors requiring careful consideration when implementing microfluidic-based sample preparation of liquid biopsy for clinical practices. Typical microfluidic-based techniques for liquid biopsy are listed in Table 1. The selection standard was mainly based on whether the technique has been transferred to commercialization or started product/kit manufacture for the already much-studied techniques such as CTC separation. As for the emerging techniques, such as bacteria and nucleated RBC, commercialization has rarely appeared, therefore the typical reports were listed. As shown in Table 1, most of the presently reported techniques are still platforms based on a single microfluidic module (i.e., single-modal microfluidics) [6,10,12,15,16,18,22,28,37,38,39], and very few of them [15,25] can satisfy all the aforementioned TSP performance criteria in clinical practices and be transferred into hospital-applicable tools. This puts forward an urgent need to bridge the gap between lab demonstrations and practical clinical applications, where integrating multiple microfluidic modules into one working system (i.e., multi-modal microfluidics, M^3^) is a promising solution, as illustrated in Figure 2. However, regarding M^3^ systems, there have not been many efforts in the procedure of sample preparation in liquid biopsy, although some related perspectives were addressed in some reviews [10,34,35]. This perspective starts a discussion about the potential approaches to develop M^3^ systems and is expected to call together researchers’ minds and efforts to combine/couple single-modal technologies for achieving successful, clinically applicable, all-in-one, sample-to-answer systems. This is believed to effectively promote the wide applications of liquid biopsy in clinics.

When implementing multiple modules into one working system, there are mainly two strategies: parallel coupling of different modules that function simultaneously (on-site M^3^) and serial combining of different modules that function separately and sequentially (in-series M^3^).

Above all, the throughput (T) is a critical index to make the time-to-result shorter and requires careful consideration for the processing of large-volume and complex heterogeneous clinical samples. Usually, a high throughput could be achieved by filtration [14,20] or multiplex microfluidic devices with the same modules (i.e., single-modal microfluidics) [12]. The throughput of M^3^ systems is influenced by multiple elements and is hard to simply calculate. Principally, for the on-site A/B M^3^, the throughput is determined by the maximum throughput of A and B, while for the in-series A-B M^3^, it will be limited by the minimum one.

The parameters of sensitivity (S) and purity (P) for a given liquid biopsy technique are usually coupled. Let us consider an M^3^ system combined two different modules (A and B). The probability density functions of target and background cell recoveries are *C_t,A_*(*D_t_*), *C_b,A_*(*D_t_*), *C_t,B_*(*D_b_*), and *C_b,B_*(*D_b_*), with *A* and *B* standing for module A and B, *t* and *b* are target cells and background cells, respectively, and *D* is the diameter of cells.

For the on-site A/B M^3^, the overall probability density functions of target and background cell recoveries *C_t,os_*(*D_t_*) and *C_b,os_*(*D_b_*) are:

(1) $$C_{t,os}\left(D_{t}\right)=1-\left[1-C_{t,A}\left(D_{t}\right)\right]\left[1-C_{t,B}\left(D_{t}\right)\right]=C_{t,A}\left(D_{t}\right)+C_{t,B}\left(D_{t}\right)-C_{t,A}\left(D_{t}\right)\times C_{t,B}\left(D_{t}\right)$$

(2) $$C_{b,os}\left(D_{b}\right)=1-\left[1-C_{b,A}\left(D_{b}\right)\right]\left[1-C_{b,B}\left(D_{b}\right)\right]=C_{b,A}\left(D_{b}\right)+C_{b,B}\left(D_{b}\right)-C_{b,A}\left(D_{b}\right)\times C_{b,B}\left(D_{b}\right)$$

From Equations (1) and (2), we will find that both the overall probability density functions for target and background cell recoveries increase compared to those of either A or B. Therefore, the on-site A/B M^3^ will improve the sensitivity (S) performance criterion. However, to improve the purity (the number of targets to the total number of targets and backgrounds after capture), we need to ensure the increment of the target probability density function (*C_t,os_*(*D_t_*) vs. *C_t,A_*(*D_t_*) or *C_t,B_*(*D_t_*)) is larger than that of background, or reduce the *C_b,A_*(*D_b_*) and *C_b,B_*(*D_b_*) as much as possible, even at the expense of target capture efficiency. One example for on-site M^3^ could be the coupling of filtration (module A) and bio-affinity (module B), i.e., antibody or aptamer functionalized micropore filter (No. 5 [16], as listed in Table 1). In practice, the bio-affinity functionalization can ensure a very low background capture efficiency (*C_b,B_*(*D_b_*)) while the combination requires that the filtration is also set *C_b,A_*(*D_b_*) as low as possible (i.e., with a larger micropore size), otherwise the background capture efficiency after combination will be dominated by the filtration one, *C_b,A_*(*D_b_*). However, if the size of the micropore is increased, the *C_t,A_*(*D_t_*) will also decrease. The decrement can be attributed not only to the apparent cell passing through but also the reduced contact between the targets and functional bio-affinity probes. In this case, cycling filtration has great potential for improving the *C_t,os_*(*D_t_*), although this may cause the working system and process to be complicated. In short, a comprehensive consideration is required to maximize the working performance and optimize the system design for simultaneously functioning multiple microfluidic modules.

Meanwhile, for the in-series A-B M^3^, the overall probability density functions of target and background cell recoveries *C_t,is_*(*D_t_*) and *C_b,is_*(*D_b_*) are:

(3) $$C_{t,is}\left(D_{t}\right)=C_{t,A}\left(D_{t}\right)\times t\times C_{t,B}\left(D_{t}\right)$$

(4) $$C_{b,is}\left(D_{b}\right)=C_{b,A}\left(D_{b}\right)\times b\times C_{b,B}\left(D_{b}\right)$$

where *t* and *b* are the coefficients of additional processing efficiency (such as releasing, transferring from module A to B, the number ratio of the output to the input) for targets and backgrounds, respectively.

From Equations (3) and (4), both the overall probability density functions for target and background cell recoveries decrease compared to those of either A or B. The good thing is that the purity (P) could be improved if the decrement of background capture efficiency is larger than that of the targets. In practice, the background capture efficiency could be expected to approach zero, such as the aforementioned bio-affinity approach, especially by a designed negative selection (e.g., anti-CD45 modified magnetic beads for WBC depletion). Again, let us take the combination of filtration and bio-affinity approaches for CTC isolation and purification as an example to discuss. As for the workflow sequence, the bio-affinity-based module can be either before or after the filtration one, theoretically, whereas the consumption of bio-affinity magnetic beads (economic cost) will be much lower if conducted after filtration, i.e., with a small number of background WBCs. However, this strategy requires a careful workflow design with a good compatibility between the two modules alongside efficient cell release and transfer, i.e., ensuring the value of *t* and *b* to be as large as possible.

To sum up, the two aforementioned strategies have revealed different advantages. The on-site M^3^ is simple to realize, but its function performance might not be easy to optimize. The different coupled modules will affect each other, which demands careful considerations and designs to optimize the overall working performance. In contrast, the in-series M^3^ may make the combination operation complicated and the working performance compromised. Nevertheless, the overall capability, especially S and P performances, can be adjusted more flexibly and easily because there are few interferences between different modules. As listed in Table 1, not all the presently tried coupling M^3^ systems are ideally effective, such as Nos. 8 [13], 10 [5], 11 [26], although still having some imperfect TSP performances. Therefore, the selection and sequence of different modules in the combination system need extensive investigations and optimization otherwise the restrictive factors (such as the low throughput of microchannel-based modules) will be inherited. It is worth mentioning that a hybrid of on-site and in-series M^3^ is also a considerable strategy to achieve liquid biopsy with optimized performance. The design of the hybrid system could be carried out by treating the system as an in-series combination of on-site M^3^ and single modal microfluidics.

In conclusion, the M^3^ systems can provide enhanced capabilities for fast-processing clinical liquid samples and the sensitive/specific separation and analysis of rare targets of interest to obtain disease-related information [4,35]. The M^3^ systems are believed to hold strong potential for commercialization and wide clinical applications. The combination itself is not the main difficulty; the key bottleneck is the proper design for both working performance guarantee and user-friendliness for widespread applications [8,19,36,37]. Besides the TSP performances, the integrity and viability of recovered targets are also important factors worth noting for expanding the liquid biopsy from detection and analysis to functional and viable characterizations, such as tumor organoid and PDX xenograft model construction for drug screening and mechanism study and therapy development. Meanwhile, the scalability (low cost) of the M^3^ systems would be crucial to securing the wide utility of microfluidic-based liquid biopsy and deploy it in hospitals for clinical diagnosis and monitoring. A lot of microfluidic-based liquid biopsy products have already been off the shelf in recent years, as shown in Table 1. However, the scale-up of manufacture in terms of material option, tubing, and adaptors to embed with standard laboratory tools and user interfaces still has a long way to realize the low-cost, single-use, and easy-to-operate employments and meet the requirements of robust and wide clinical applications. Last but not least, a thoughtful partnership (i.e., intelligence integration) between academia and industry is worth mentioning. The engineers working on M^3^ -based liquid biopsy techniques should engage in more thoughtful and meaningful partnerships with biologists/clinicians and industry research scientists/market investigators. The intelligence integration will increase the practical applicability and robustness of engineers’ findings and inventions and realize the widespread adoption by the end-users (academic biologists, industry research scientists, and clinicians) for ultimate applications and practices.

## Figures and Tables

**Figure 1:**
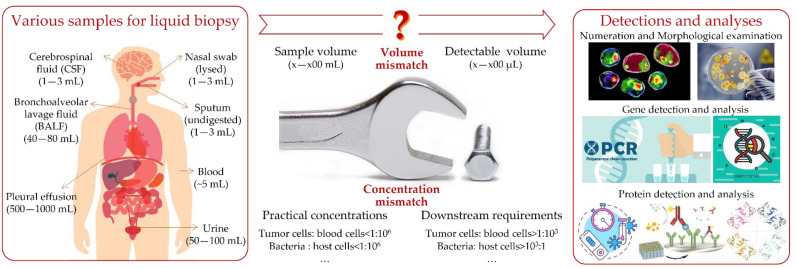
Schematic illustration of the gap between the raw samples and detection/analysis platforms in the practices of liquid biopsy. All the images used here were commercially bought from iStock (https://www.istockphoto.com) on 23 January 2022 with issued copyright for reprint.

**Figure 2:**
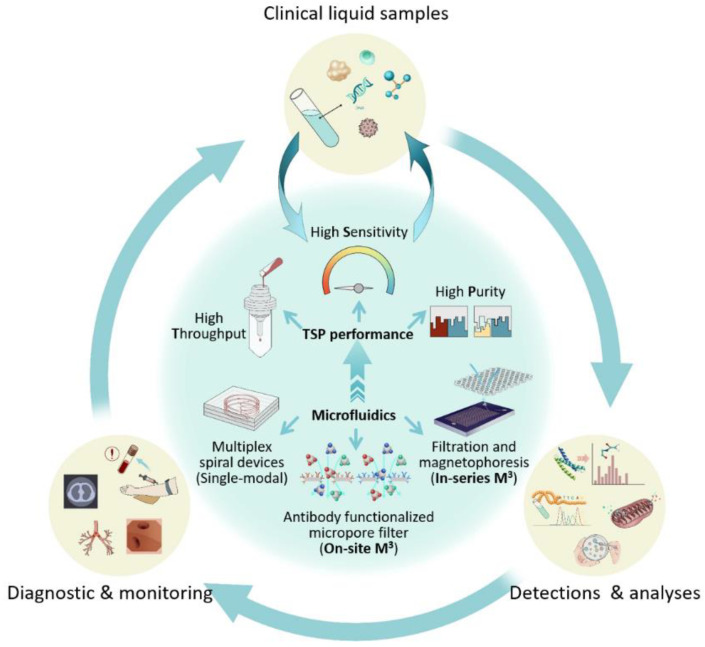
Schematic illustration of clinical practices of liquid biopsy, including the TSP performance criteria and the potential microfluidic-based strategies [10,12,14,16,34,35,38]. All the images/icons displayed here were drawn and are fully copyrighted by authors.

**Table 1:** List of typical microfluidic-based techniques for liquid biopsy.

No.	Targets of Interest	Working Principle	Modal Category	TSP Performance			Sample	Product/ Company	Ref.
				Throughput ^#1^	Sensitivity ^#2^	Purity ^#3^			
1	CTC	Bio-affinity	Single-modal	Low	High	High	Blood	CTC-Chip/ Veridex LLC *^1^	[18]
2	CTC	Size differences (Dean flow fractionation)	Single-modal	Moderate–High	High	Moderate	Blood	ClearCell^®^ FX system/ Biolidics *^2^	[12]
3	CTC	Bio-affinity (magnetophoresis)	Single-modal	Low	High	High	Blood	LiquidBiopsy^®^/ Cynvenio *^3^	[17,21]
4	CTC/ ETC/ Fungi	Size differences (filtration)	Single-modal	High	High	Moderate–High	Blood BALF Urine	PERFECT filter/ Branemagic *^4^	[20,21,25]
	CTC		Single-modal				Blood	CTC enumeration/ VyCAP *^5^	[15]
5	CTC	Size differences (filtration) and bio-affinity	Multi-modal (on-site M^3^)	High	High	High	Blood	N/A	[16]
6	CTC/ WBC/ Bacteria/ Exosome	Size differences (acoustophoresis)	Single-modal	Low–Moderate	Moderate–High	Moderate	Blood	ACOUTRAP, ACOUWASH/ AcouSort *^6^	[39]
8	Nucleated RBC	Size differences (deterministic lateral displacement) and bio-affinity	Multi-modal (on-site M^3^)	Low–Moderate	High	High	Blood	FETAL-Chip/ Wisdom healthy *^7^	[13]
9	Bacteria	Size differences (elasto-inertial microfluidics)	Single-modal	Low–Moderate	Moderate–High	Moderate	Blood	N/A	[6]
10	Bacteria	Size differences (filtration) and bio-affinity (magnetophoresis)	Multi-modal (in-series M^3^)	Moderate–High	Moderate–High	High	Blood	N/A	[5]
11	Bacteria	Electrical property (dielectrophoresis) and bio-affinity (magnetophoresis)	Multi-modal (on-site M^3^)	Low	High	High	Buffer	N/A	[26]
12	Exosome	Size differences (e.g., filtration, deterministic lateral displacement, acoustophoresis) or bio-affinity	Single-modal	Low–Moderate	Moderate–High	Moderate	Blood Urine CSF Saliva	Creative Biolabs^®^ Exosome *^8^	[32]

The presently reported typical microfluidic techniques listed in Table 1 are arranged based on the size of the targets of interest (from micrometer-sized cells to sub-micrometer-sized bacteria and exosome), and then the working principle from single- to multi-modal microfluidics for the same sized targets. *^1^ http://www.veridex.com/; *^2^ https://www.biolidics.com/; *^3^ https://www.cynvenio.com/, http://www.sanmedbio.com/gywm; *^4^ www.branemagic.com; *^5^ https://www.vycap.com/; *^6^ https://acousort.com/; *^7^ http://www.dykm-biotech.com/; *^8^ https://www.creative-biolabs.com/exosome/exosome-isolation.htm; ^#1^ The clarification standard of “Throughput” (T) performance is: T ≤ 0.1 mL/min (Low), 0.1 mL/min < T ≤ 1 mL/min (Moderate), T > 1 mL/min (High). ^#2^ and ^#3^ The parameters used to claim the “Sensitivity” (S) and “Purity” (P) performance are different in the reported works. Taking the liquid biopsy of blood as an example, the sensitivity related parameters mainly include the recovery rate of spiked cells in demo samples and the so-called limit of detection of tumor cells in clinical samples. The purity-related parameters mainly include the real purity (ratio of no. of captured CTCs to the total no. of captured CTCs and WBCs) and depletion efficiency of WBCs. Therefore, it is hard to display the numerical information for easy/obvious comparison and the performance clarifications were carefully identified according to the working principle and listed with the modes of “Low”, “Moderate”, and “High”.

## Data Availability

The data that support the findings of this study are available from the corresponding author upon reasonable request.
